# Effect of Residual CaSO_4_ in Clinker on Properties of High Belite Sulfoaluminate Cement Based on Solid Wastes

**DOI:** 10.3390/ma13020429

**Published:** 2020-01-16

**Authors:** Dunlei Su, Qiuyi Li, Yuanxin Guo, Gongbing Yue, Liang Wang

**Affiliations:** 1 School of Civil Engineering, Qingdao University of Technology, Qingdao 266033, China; sudunlei@163.com; 2 School of Architectural Engineering, Qingdao Agricultural University, Qingdao 266109, China; yuegongbing@163.com (G.Y.); jiangongwl_2019@163.com (L.W.)

**Keywords:** residual CaSO_4_, solid wastes, high belite sulfoaluminate cement, petroleum coke desulfurization slag, CaSO_4_ type, CaSO_4_ content, cement properties

## Abstract

The high belite sulfoaluminate cement (HBSAC) containing CaSO_4_, and without CaSO_4_, based on solid wastes were successfully prepared from petroleum coke desulfurization slag (PCDS), fly ash (FA), carbide slag (CS), and bauxite (BX). The mineral composition of clinkers after different calcination history were investigated by X-ray fluorescence (XRF), X-ray diffraction (XRD)/Quantitative X-ray diffraction (QXRD), and scanning electron microscopy (SEM), so as to determine the calcination temperatures. The difference between residual CaSO_4_ and dihydrate gypsum (DG) and the optimal content of residual CaSO_4_ were discussed by studying the properties of HBSAC. The results revealed that the residual CaSO_4_ in clinker could replace DG to participate in hydration, and showed some advantages in strength and early hydration heat, but meanwhile increased the water requirement of normal consistency and hydration heat at 72 h, and prolonged the setting time. With the increase of residual CaSO_4_ content in clinker, the lower limit temperature of clinker formation gradually increased, and the crystal size of clinker minerals became finer and the boundary between crystals became more blurred. However, the optimal calcination temperature (1300 °C) of HBSAC clinker did not change. Considering the effect of residual CaSO_4_ content on the water requirement of normal consistency, setting time, hydration heat, strength, and hydration products, the optimal design content of residual CaSO_4_ in HBSAC clinker based on solid wastes, such as PCDS and FA, was 15%.

## 1. Introduction

As an inorganic nonmetallic material with obvious advantages, such as high early strength, good later strength development, low energy consumption, and CO_2_ emission, high belite sulfoaluminate cement (HBSAC) has become a hot spot in the field of sulfoaluminate cement (SAC). The mineral components of this cement clinker are mainly C_4_A_3_$\bar{S}$ (3CaO·3Al_2_O_3_·CaSO_4_) and β-C_2_S (2CaO·SiO_2_). The raw materials for production are usually limestone, clay, bauxite, and gypsum [1,2]. However, with the long-term and large-scale exploitation of natural resources, the above-mentioned raw materials are becoming more and more scarce, and all kinds of industrial solid wastes are beginning to be used as alternative raw materials, including calcium solid waste materials such as marble sludge waste [3], steel slag [4,5], lithium slag [6] and municipal solid incineration wastes [7], sulfur solid waste materials such as phosphogypsum [8,9] and desulfurization gypsum [10,11], and aluminum–silicon solid waste materials such as fly ash (FA) [11,12,13,14,15,16], coal gangue [17], red mud [10,18], aluminum anodizing sludge [19], and tailings [20]. C_4_A_3_$\bar{S}$ is an indispensable component in the hydration of HBSAC, and the main chemical reactions in the hydration process are shown in Equations (1) and (2). C_4_A_3_$\bar{S}$ can be hydrated to form AFt (C_3_A·3C$\bar{S}$·32H) under the condition of sufficient CaSO_4_, while C_4_A_3_$\bar{S}$ can be hydrated to form AFm (C_3_A·C$\bar{S}$·12H) under the condition of insufficient CaSO_4_. Compared with AFm, AFt has higher strength and plays a more important role in the development of cement strength, so it seems to be a reasonable choice to have some residual CaSO_4_ in clinker, which can not only reduce the amount of natural gypsum, but also greatly improve the utilization rate of sulfur solid waste materials.

(1) $$C_{4}A_{3}\bar{S}\;+\;2C\bar{S}H{}_{2}+\;34H\;\to \;C_{3}A\cdot 3C\bar{S}\cdot 32H\;(\mathrm{AFt})\;+\;2\mathrm{AH}{}_{3}(\mathrm{gel}).$$

(2) $$C_{4}A_{3}\bar{S}\;+\;18H\;\to \;C_{3}A\cdot C\bar{S}\cdot 12H\;(\mathrm{AFm})\;+\;2\mathrm{AH}{}_{3}(\mathrm{gel}).$$

The preparation of HBSAC clinker containing CaSO_4_ from solid wastes has been studied before. Chen et al. [14] successfully prepared HBSAC clinker with mineral content of about 43% C_4_A_3_$\bar{S}$, 42% C_2_S, 8% C_4_AF (4CaO·Al_2_O_3_·Fe_2_O_3_), and 3% CaSO_4_ from industrial wastes such as FA, flue gas desulfurization sludge, and fluidized bed ash. Lv et al. [21] prepared HBSAC clinker with mineral content of about 20% C_4_A_3_$\bar{S}$, 56% C_2_S, 17% C_2_AF (2CaO·Al_2_O_3_·Fe_2_O_3_), and 3.5% CaSO_4_ by using limestone, bauxite (BX), and ashes from circulating fluidized bed combustion. However, the residual CaSO_4_ content in both clinkers was low, and it was often necessary to admix a certain amount of natural gypsum with the clinker to make the cement achieve better performance, which could not save the natural gypsum resources to a large extent. For example, HBSAC clinker prepared by Chen et al. needed 20% natural dihydrate gypsum (DG) to achieve stable exothermic rate, but the cement pastes exhibited expansion cracking under this condition, which needed 1% citric acid retarder to eliminate. When 20% natural DG and 1% citric acid retarder were added, the compressive strength of cement mortar prepared according to ASTM C 109 standard could reach 15, 24, 29, and 45 MPa at 1, 3, 7, and 28 days, respectively. As for HBSAC clinker prepared by Lv et al., the compressive strength of cement pastes under the condition of water:cement ratio of 0.3 could reach 25 and 45 MPa at 3 and 28 days, respectively. When 10% natural DG was added, the compressive strength of cement pastes could reach the maximum value, which was 35 and 80 MPa at 3 and 28 days, respectively. 

Tangshan Polar Bear Building Materials Co., Ltd. [11] has produced HBSAC clinker with mineral composition of 20%–35% C_4_A_3_$\bar{S}$, 37%–47% C_2_S, 3%–9% C_4_AF, 0.5%–4.6% f-CaO, and 14%–26.3% CaSO_4_ by using FA, desulfurization gypsum, and other natural raw materials. This clinker could be directly used as cement, and showed many advantages, such as fast setting and hardening, early strength and high strength, low shrinkage, impermeability, frost resistance, corrosion resistance, and so on. Nowadays, the relevant production technology of this clinker has been relatively perfect, and good social benefits have been achieved in the engineering applications of Qingdao Airport and oilfields of Dongying. Taking HBSAC clinker with mineral composition of 28.6% C_4_A_3_$\bar{S}$, 39.6% C_2_S, 4.6% C_4_AF, 1.6% f-CaO, and 13.6% CaSO_4_ as an example, its water requirement of normal consistency was 31.3%, and its initial and final setting time were 9 and 13 min, respectively. The compressive strength of cement mortar prepared under the condition of water:cement ratio of 0.5 could reach over 20 MPa at 4 h, 30 MPa at 1 day, and 45 MPa at 28 days. At the same curing age, the bending strength could reach 4.3, 5.1, and 7.6 MPa, respectively. The cumulative heat released from hydration at 3 days was approximately 280 J/g. When mixed with anhydrite in cement clinker, the anhydrite content had different effect on various cement properties. With the increase of anhydrite content, the setting time did not change much and the water requirement of normal consistency decreased. In terms of compressive strength, the early compressive strength increased and the later strength decreased under the condition of 10% anhydrite, while the early compressive strength decreased and the later strength increased under the condition of 20% anhydrite. 

Shen et al. [8] used phosphogypsum, FA, and other natural raw materials to prepare a series of HBSAC clinkers with residual CaSO_4_ content ranging from 0% to 20%. Moreover, the other mineral composition of these clinkers were approximately 25%–40% C_4_A_3_$\bar{S}$, 30%–65% C_2_S, and 10% C_4_AF. The residual CaSO_4_ in clinker could achieve similar effect to that of additional natural gypsum, and was even better than additional natural gypsum in cement properties. Meanwhile, the residual CaSO_4_ content in clinker also had different effect on cement properties. In the aspect of setting time, there was little difference when the residual CaSO_4_ content was lower than 10%, and there was an obvious growth trend when the residual CaSO_4_ content was higher than 10%. The strength increased gradually with the increase of residual CaSO_4_ content, while the expansion rate did not change obviously, and the strength increased most significantly when the residual CaSO_4_ was 10%. For HBSAC clinker with mineral composition of 35.0% C_4_A_3_$\bar{S}$, 32.3% C_2_S, 8.4% C_4_AF, and 16.6% CaSO_4_, when the water:cement ratio was 0.5, the initial and final setting time of cement paste were 130 and 220 min, the compressive strength of cement paste could reach 25, 30, 36, and 38 MPa at 1, 3, 7, and 28 days, respectively. At the same time, the cumulative heat released from hydration at 1 day reached 210 J/g. The expansion rate of cement mortar was basically stable at 1.5 × 10^−4^ according to the “Standard test method for drying shrinkage of mortar” (JC/T 603-2004, China). 

It was not difficult to find that remarkable achievements had been made in the preparation of HBSAC clinker containing CaSO_4_ from solid wastes, but there were still some problems to be solved urgently. Firstly, the method of improving cement properties by adding gypsum into HBSAC clinker with low CaSO_4_ content not only wasted natural resources, but also brought more uncertainty to cement hydration because the effect of adding gypsum was not completely equal to the residual CaSO_4_ in clinker. Secondly, even though HBSAC clinker with high CaSO_4_ content could show excellent properties without adding gypsum, the properties of HBSAC clinker prepared with different solid wastes were also quite different, such as HBSAC clinkers prepared by Tangshan Polar Bear Co., Ltd. (Tangshan, China) and Shen et al. Petroleum coke desulfurization slag (PCDS) is the residue of sulfur-containing petroleum coke and desulfurizer (usually limestone) calcined at high temperature in a circulating fluidized bed boiler, which is composed of CaO and CaSO_4_. It can not only replace the limestone which produces a lot of CO_2_ and the natural gypsum which is in short supply, providing calcium and sulfur elements needed for the formation of clinker minerals such as C_4_A_3_$\bar{S}$ and C_2_S, but also provide raw materials for residual CaSO_4_ in clinker. In this study, PCDS, FA, carbide slag (CS), and BX were used together to prepare HBSAC clinker, including some containing different CaSO_4_ contents and some without CaSO_4_. At the same time, the effect of residual CaSO_4_ content on calcination temperature of clinkers was discussed. Based on the study of cement properties, the effect of residual CaSO_4_ in clinker and DG were compared, and the optimal content of residual CaSO_4_ was determined, which would provide theoretical basis for the reduction and absorption of solid wastes, such as petroleum coke desulfurization slag, and the proportional optimum design of HBSAC based on solid wastes.

## 2. Experiment

### 2.1. Raw Materials

**Figure 1:**
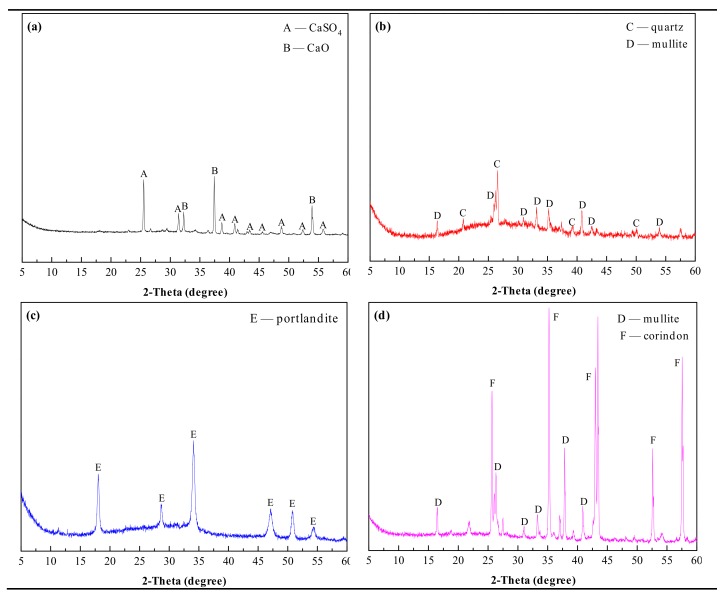
XRD patterns of raw materials: (**a**) PCDS; (**b**) FA; (**c**) CS; (**d**) BX.

In the experiment, PCDS was taken from the Sinopec Qingdao Refining and Chemical Co., Ltd. (Qingdao, China), FA (Class I) from the Qingdao Municipal Concrete Industry Co., Ltd. (Qingdao, China), CS from the Qingdao Qingxin Building Materials Co., Ltd. (Qingdao, China), BX from the Gongyi Wanying Environmental Protection Materials Co., Ltd. (Gongyi, China), and DG from the Sinopharm Chemical Regent Co., Ltd. (Shanghai, China). The main chemical compositions of raw materials are listed in Table 1, and the main mineral compositions of PCDS, FA, CS, and BX are shown in Figure 1.

### 2.2. Materials Proportioning

Based on the ternary mineral system of SAC clinker containing C_4_A_3_$\bar{S}$, C_2_S and C_4_AF, CaSO_4_ was introduced into the mineral system of new clinker. Meanwhile, considering that the C_2_S content in HBSAC clinker is generally more than 40%, the mineral composition of the clinkers and mixing design of raw materials are shown in Table 2. The Series GF-HBSAC-X cement was used to study the effect of CaSO_4_ type on the properties of HBSAC based on solid wastes, and the Series GF-HBSAC-Y cement was used to study the effect of residual CaSO_4_ content on the properties of HBSAC based on solid wastes.

**Figure 2:**
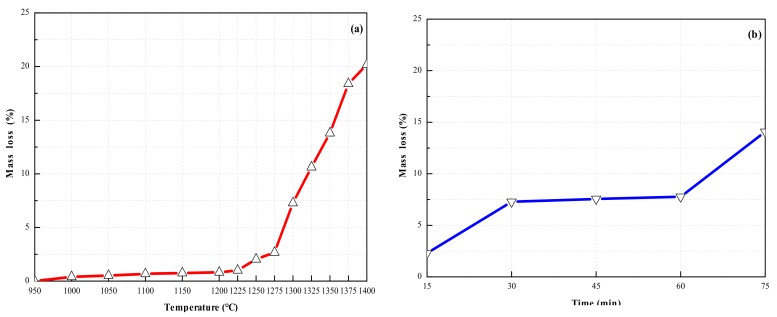
Mass loss of PCDS (**a**) at different calcination temperatures; (**b**) for different calcination time.

In the process of developing a mixing design, the design principles are as follows: (1) The mineral composition of clinker is preliminarily set up, assuming that the following three reactions occur during calcination: 4CaO + Al_2_O_3_ + Fe_2_O_3_ → C_4_AF, 3CaO + 3Al_2_O_3_ + CaSO_4_ → C_4_A_3_$\bar{S}$, 2CaO + SiO_2_ → C_2_S, and the amount of raw materials was deduced according to Table 1. (2) As a raw material of calcium and sulfur, PCDS contains high CaSO_4_ content. CaSO_4_ is decomposed by heat in high temperature reaction, which will affect the amount of PCDS. After being calcined to a constant weight at 950 °C, the mass loss of PCDS calcined at higher temperatures is shown in Figure 2. Figure 2a shows the mass loss of PCDS calcined at different temperatures for 30 min. When the temperature is not higher than 1275 °C, the mass loss is less than 2.5%. When the temperature is 1300 °C, the mass loss is approximately 7.5%. When the temperature reaches 1325–1350 °C, the mass loss is about 12.5%, and when the temperature is not lower than 1375 °C, the mass loss is nearly 20%. Figure 2b shows the mass loss of PCDS calcined for different times at 1300 °C. When PCDS is calcined for 15 min, the mass loss is about 2.5%. When PCDS is calcined for 30–60 min, the mass loss is basically maintained at 7.5%. When PCDS is calcined for 75 min, the mass loss is close to 15%. In this study, the calcination time was fixed at 30 min, and additional PCDS was added to the proportion scheme according to the data in Figure 2a at each calcination temperature. (3) In the production of SAC, the three moduli (alkalinity coefficient: *C_m_*, Al:S ratio: *P* and Al:Si ratio: *N*) [22] is usually used to control the clinker composition and adjust materials proportioning. The cement clinker involved in this study is HBSAC clinker, the C_2_S content is usually higher than C_4_A_3_$\bar{S}$, and most of the clinkers contain residual CaSO_4_, so *C_m_* was designed to be slightly larger than 1.0, *P* not more than 3.82, and *N* not controlled.

### 2.3. Preparation Process

**Figure 3:**
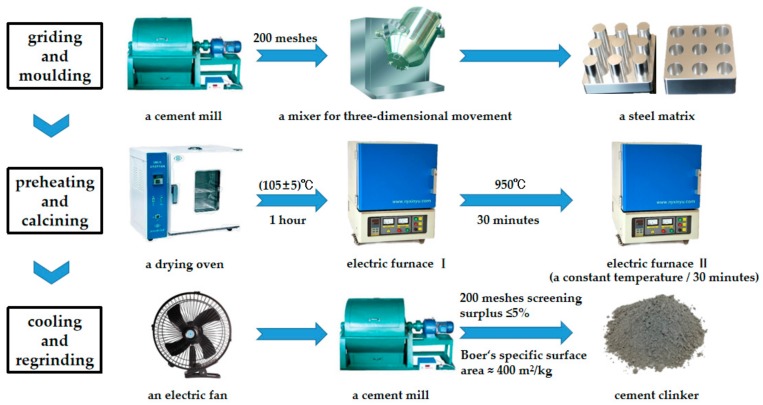
Preparation process of HBSAC clinker based on solid wastes.

The preparation of HBSAC clinker based on solid wastes was mainly divided into three steps: Grinding and molding, preheating and calcining, and cooling and regrinding, as shown in Figure 3. 

Grinding and molding: All of the raw materials were ground by a cement mill to pass through a 200 mesh square hole sieve, and then mixed evenly by a mixer for three-dimensional movement, and finally pressed into a steel matrix to form cylindrical specimens of φ × h = 15 × (15 – 20) mm.

Preheating and calcining: The samples were dried for 1 h in a drying oven at 105 ± 5 °C, and then preheated for 30 min in a high temperature electric furnace at 950 °C, and again calcined for 30 min in another high temperature electric furnace at a constant temperature.

Cooling and regrinding: The samples were taken out from the high temperature electric furnace, and then cooled by an electric fan, and finally ground to pass through a 200 mesh square hole sieve with a surplus of less than 5% or a Boer’s specific surface area of about 400 m^2^/kg.

### 2.4. Test Methods

-XRF: An X-ray fluorescence spectrometry (1800 type, Shimadzu Co., Kyoto, Japan) was used to analyze the chemical composition of the raw materials and clinkers. The test results were given in terms of oxide content.

-XRD: An X-ray diffraction instrument (D8 advance type, Bruker Co., Karlsruhe, Germany) was applied to detect mineral composition of the clinkers and hydration products. Its working conditions were as follows: Cu target, voltage at 40 kV, current at 40 mA, 2-Theta scanning ranges from 5° to 60°, step width of 0.02°, and a residence time of 0.05 s. In order to quantify phases of the clinkers, QXRD analysis was conducted by using EXPGUI-GSAS [23] software to refine XRD patterns of the clinkers. It should be noted that all samples were in powder form, and the samples of hydration products were obtained from 20 × 20 × 20 mm cement pastes prepared under the condition of water:cement ratio of 0.50.

-SEM: A scanning electron microscopy (JSM-7500F type, JEOL Co., Ltd., Tokyo, Japan) was adopted to characterize the micromorphology of the clinker minerals and hydration products with a working voltage of 2.0 kV. The samples of the clinkers were in powder, and the samples of the hydration products were in pieces, which were all sprayed with gold before observed. The samples of hydration products were also obtained from 20 × 20 × 20 mm cement pastes prepared under the condition of water:cement ratio of 0.50.

-Isothermal calorimetric: An eight-channel isothermal conduction calorimeter (TAM Air type, Thermometric AB Co., Jafalla, Sweden) was used to measure the hydration heat of cements. In the test, the temperature was set at 25 °C, the water:cement ratio was fixed to 0.5, and the hydration heat were recorded over a period of 72 h.

-Thermal analysis: A comprehensive thermal analyzer (SDT Q600 type, TA Instruments Co., New Castle, DE, USA.) was used to conduct differential thermal analysis of hydration products. The temperature varied from room temperature (approximately 20 °C) to 800 °C at a heating rate of 20 °C/min with a flux of N_2_. The samples of the hydration products were in powder, and the samples of hydration products were the same as the samples used for XRD analysis.

-Particle size distribution: A laser particle size analyzer (Rise-2006 type, Jinan Rise Science and Technology Co., Ltd., Jinan, China) was adopted to test the particle size distributions of cements or clinkers and alcohol was selected as dispersing medium. 

-Physical and mechanical properties test: The water requirement of normal consistency and setting time were determined according to the “Test methods for water requirement of normal consistency, setting time and soundness of the Portland cement” (GB/T 1346-2011, China). The mechanical strength was tested according to the “Method of testing cements-Determination of strength” (GB/T 17671-1999, China) and the “sulfoaluminate cement” (GB 20472-2006, China). The size of cement mortars were 40 × 40 × 160 mm, and the water requirement was determined by controlling the fluidity to 170 mm. The size of cement pastes were 20 × 20 × 20 mm, which were prepared under the condition of water:cement ratio of 0.35 and 0.50. After preparation, all samples were placed in a curing room with a temperature of 20 ± 1 °C and a relative humidity of no less than 90% for 6 h, then demolded and cured in water until different ages. 

## 3. Results and Discussion

### 3.1. Preparation of HBSAC Based on Solid Wastes

#### 3.1.1. Series GF-HBSAC-X Cement

**Figure 4:**
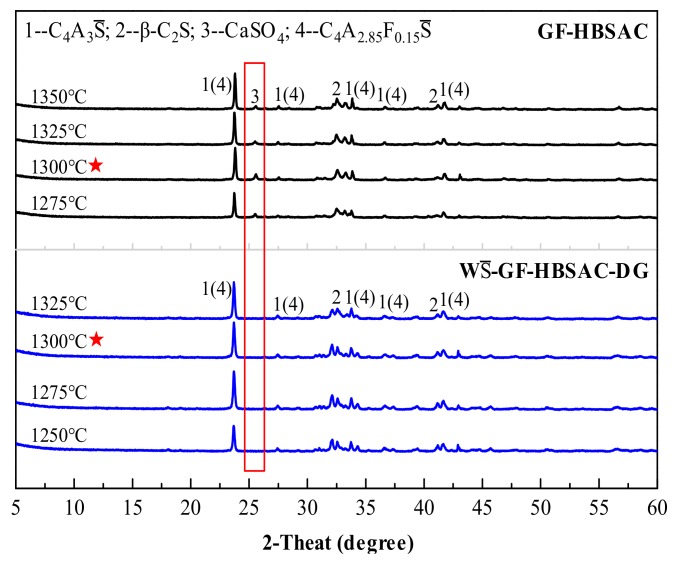
X-ray diffraction (XRD) patterns of the Series GF-HBSAC-X cement clinkers at different temperatures.

Figure 4 shows the XRD patterns of GF-HBSAC and W$\bar{S}$-GF-HBSAC-DG clinkers at different calcination temperatures. It can be seen from the figure that there are obvious CaSO_4_ diffraction peaks in the XRD patterns of GF-HBSAC clinker, while there was no CaSO_4_ diffraction peaks in the XRD patterns of W$\bar{S}$-GF-HBSAC-DG clinker, which is consistent with the designed mineral composition of two clinkers. In addition, both clinkers contain minerals such as C_4_A_3_$\bar{S}$, β-C_2_S and C_4_A_2.85_F_0.15_$\bar{S}$, among which C_4_A_3_$\bar{S}$ and β-C_2_S are the main mineral components of SAC, and C_4_A_2.85_F_0.15_$\bar{S}$ is a solid solution formed by the solid solution of iron element in C_4_A_3_$\bar{S}$. According to the relationship between the intensity of the diffraction peaks, the optimal calcination temperature of the two clinkers is 1300 °C.

For each of two clinkers prepared at 1300 °C, the chemical composition was tested by XRF, and the three moduli was calculated. The results are all listed in Table 3. It is worth noting that the equations for calculating the three moduli should be adjusted because of the existence of C_4_A_2.85_F_0.15_$\bar{S}$ and CaSO_4_ in clinker, and the adjusted equations are as follows (3)–(5).

*C_m_* = {ω(CaO) − 0.7ω(TiO_2_) − 1.70[ω(SO_3_) − 0.26ω(Al_2_O_3_)]}/[0.73ω(Al_2_O_3_) + 1.87ω(SiO_2_)]. (3)

*P* = ω(Al_2_O_3_)/ω(SO_3_). (4)

*N* = ω(Al_2_O_3_)/ω(SiO_2_). (5)

**Figure 5:**
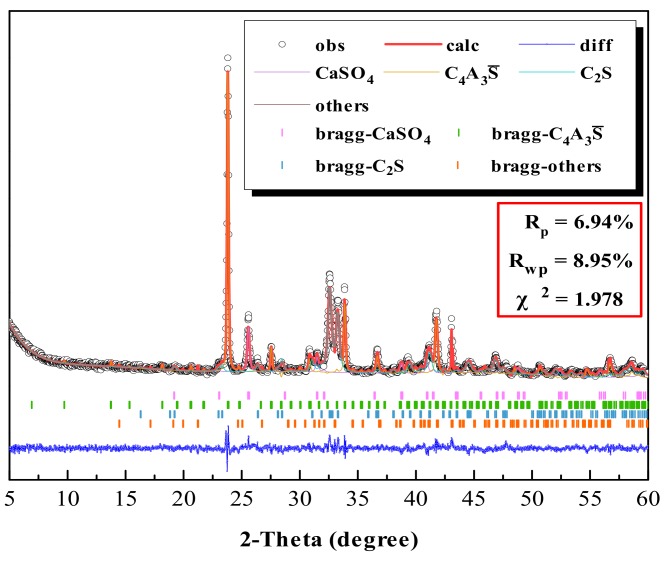
Refined X-ray diffraction (XRD) pattern of GF-HBSAC clinker by using GSAS.

The mineral content of two clinkers was analyzed by QXRD [19,24,25] using GSAS software. The results are listed in Table 4. Comparing Table 4 and Table 2, the actual mineral content of each clinker is close to the designed mineral content, and the content error is within a reasonable range. Figure 5 shows the refined XRD pattern of GF-HBSAC clinker. As can be seen from this figure, the fitting peak shape (surrounded by black circle) is similar to the experimental peak shape (surrounded by red solid line), and R_wp_ = 8.95 < 10 and χ^2^ = 1.978, so it can be considered that the refined result is reliable. For this reason, the reliability of materials proportioning and calcination system has also been verified.

**Figure 6:**
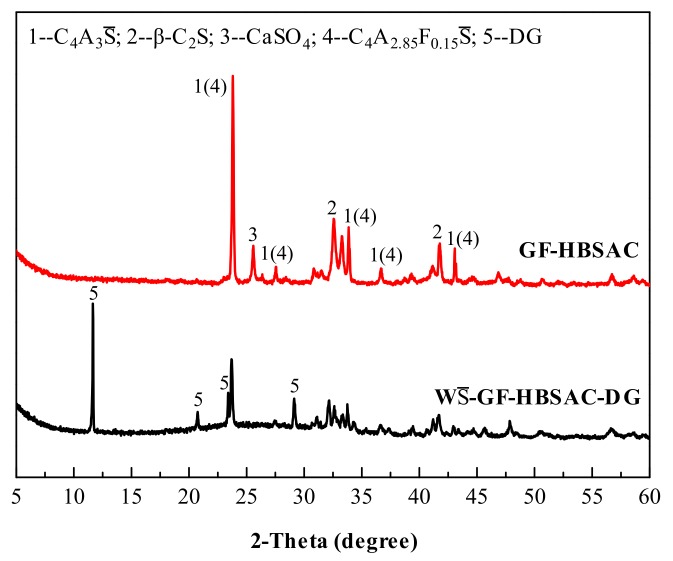
X-ray diffraction (XRD) patterns of the Series GF-HBSAC-X cement.

**Figure 7:**
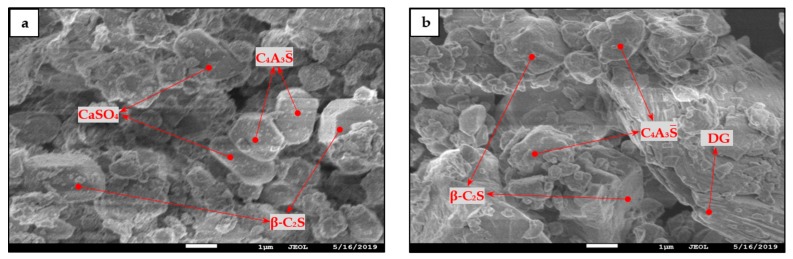
SEM micromorphology of the Series GF-HBSAC-X cement: (**a**) GF-HBSAC cement; (**b**) W$\bar{S}$-GF-HBSAC-DG cement.

In combination with the results of QXRD analysis, W$\bar{S}$-GF-HBSAC-DG cement was prepared by mixing 84% W$\bar{S}$-GF-HBSAC-DG clinker with 16% DG on the principle of equal CaSO_4_ and C_4_A_3_$\bar{S}$ contents in GF-HBSAC and W$\bar{S}$-GF-HBSAC-DG cement. Figure 6 shows the XRD patterns of the two cements. It can be seen from the figure that there are obvious diffraction peaks of β-C_2_S and C_4_A_3_$\bar{S}$ (C_4_A_2.85_F_0.15_$\bar{S}$) in both cements, but the diffraction peaks of CaSO_4_ component show that the CaSO_4_ component in GF-HBSAC cement is anhydrous CaSO_4_, while the CaSO_4_ component in W$\bar{S}$-GF-HBSAC-DG cement is DG. Figure 7 shows the SEM micromorphology of the two cements. In the two cements, blocky and granular β-C_2_S and polygonal and tabular C_4_A_3_$\bar{S}$ are all observed, and the obvious difference lies in the morphology of CaSO4 component. The CaSO_4_ component in GF-HBSAC cement is rectangular while the CaSO_4_ component is lamellar in W$\bar{S}$-GF-HBSAC-DG cement, which is consistent with the crystal structure of anhydrous CaSO_4_ and DG [25,26,27,28,29].

#### 3.1.2. Series GF-HBSAC-Y Cement

**Figure 8:**
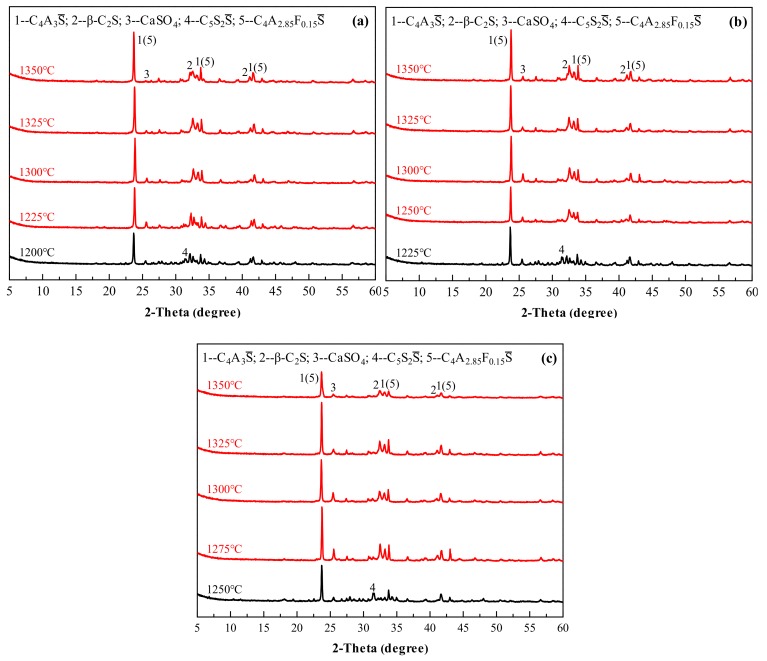
X-ray diffraction (XRD) patterns of the Series GF-HBSAC-Y cement clinkers at different temperatures: (**a**) GF-HBSAC-10%; (**b**) GF-HBSAC-15%; (**c**) GF-HBSAC-20%.

Figure 8 shows the XRD patterns of the Series GF-HBSAC-Y cement clinkers prepared at different calcination temperatures. It is; thus, clear that the formation temperature of cement clinkers with coexisting minerals, including C_4_A_3_$\bar{S}$, β-C_2_S, and CaSO_4_, is affected by residual CaSO_4_ content in clinker. For GF-HBSAC-10% clinker, GF-HBSAC-15% clinker, and GF-HBSAC-20% clinker, the formation temperature ranges from 1225 to 1350 °C, 1250 to 1350 °C, and 1275 to 1350 °C, respectively. That is to say, with the increase of residual CaSO_4_ content in clinker, the lower limit temperature of clinker formation gradually increases. The reason for this phenomenon lies in the formation and decomposition of C_5_S_2_$\bar{S}$ [15,19,30], and the highest temperature of C_5_S_2_$\bar{S}$ in the above three clinkers reaches 1200, 1225, and 1250 °C, respectively. For the optimal calcination temperature and the upper limit temperature of clinker formation, there is little difference among the three clinkers. According to the relationship between the intensity of the diffraction peaks, the optimal calcination temperature of the three clinkers is 1300 °C. It is reasonable to set the upper limit temperature at 1350 °C, at which the decomposition of various minerals has been very large, especially the CaSO_4_.

For each of the three clinkers prepared at 1300 °C, the chemical composition was tested by XRF, and the three moduli was calculated according to Equations (3)–(5), and the results are listed in Table 5. The mineral content of the three clinkers was still analyzed by QXRD using GSAS software and the results are listed in Table 6.

**Figure 9:**
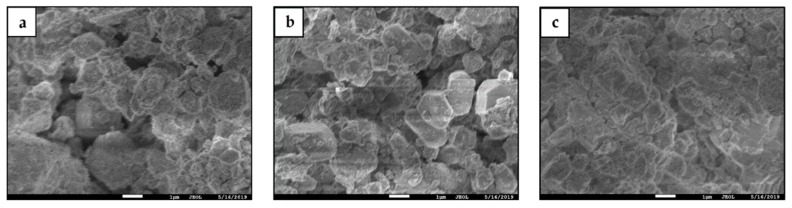
SEM micromorphology of the Series GF-HBSAC-Y cement: (**a**) GF-HBSAC-10%; (**b**) GF-HBSAC-15%; (**c**) GF-HBSAC-20%.

Figure 9 shows SEM micromorphology of the Series GF-HBSAC-Y cement clinkers. In general, the mineral micromorphology of the three clinkers seems to be the same, but there are some differences in detail. As shown in Figure 9a, the boundary between crystals is obvious and the crystal size of clinker minerals is large. In addition, in Figure 9b, the boundary between crystals is still obvious but the crystal size of clinker minerals becomes smaller; and in Figure 9c, the boundary between crystals is no longer clear, which is consistent with the results of reference [31].

### 3.2. Effect of CaSO_4_ Type on Properties of HBSAC Based on Solid Wastes

#### 3.2.1. Effect of CaSO_4_ Type on Water Requirement of Normal Consistency and Setting Time

**Figure 10:**
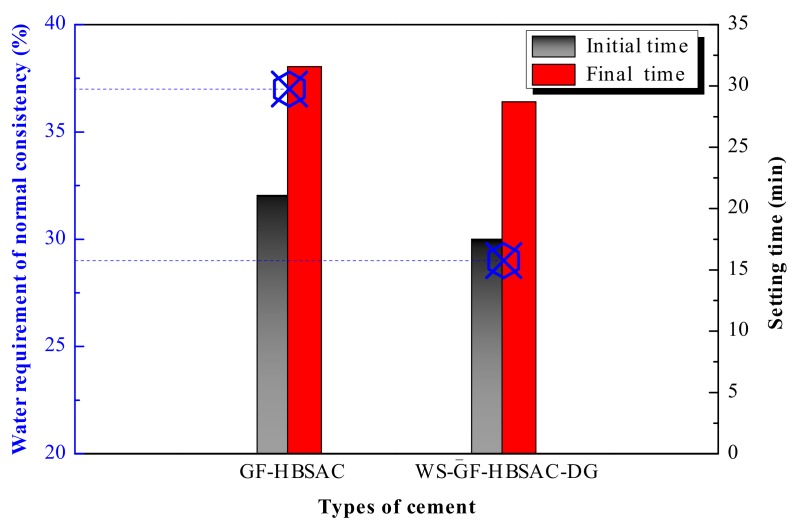
Water requirement of normal consistency and setting time of the Series GF-HBSAC-X cement.

As shown in Figure 10, the water requirement of normal consistency of GF-HBSAC cement reaches 37%, whereas that of W$\bar{S}$-GF-HBSAC-DG cement is only 29%. The main reason for the obvious difference is that the residual CaSO_4_ in GF-HBSAC clinker dissolves, diffuses, and crystallizes into DG after encountering water, resulting in an increase in water demand. In terms of setting time, the setting time of GF-HBSAC cement is longer than that of W$\bar{S}$-GF-HBSAC-DG cement, which is attributed to the different hydration activity of residual CaSO_4_ in clinker and DG. Studies in [32,33] have shown that DG and hemihydrate gypsum can more effectively promote the dissolution of C_4_A_3_$\bar{S}$ and the formation of AFt than anhydrous CaSO_4_, and the reason is that slow dissolution of anhydrous CaSO_4_ leads to insufficient supply of calcium and sulfate ions.

#### 3.2.2. Effect of CaSO_4_ Type on Hydration Heat

**Figure 11:**
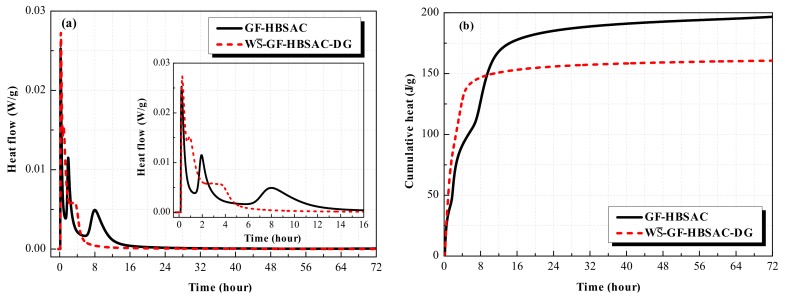
Hydration heat curves of the Series GF-HBSAC-X cement: (**a**) Heat flow curve; (**b**) cumulative heat curve.

**Figure 12:**
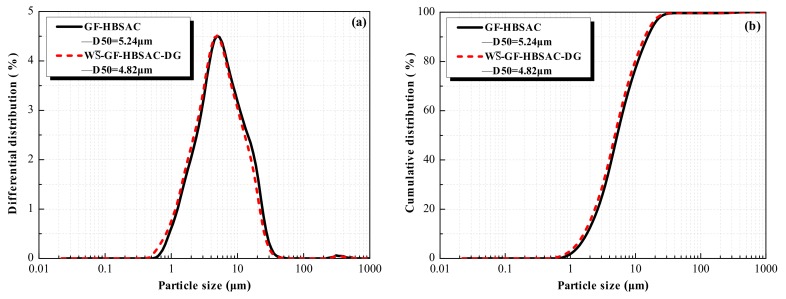
Particle size distribution curves of the Series GF-HBSAC-X cement: (**a**) Differential distribution; (**b**) cumulative distribution.

Figure 11 shows the hydration heat curves of the Series GF-HBSAC-X cement. As can be seen from Figure 11a, there are three exothermic peaks in the heat flow curve of GF-HBSAC cement and W$\bar{S}$-GF-HBSAC-DG cement, which is the same as the findings in [19]. The first exothermic peak is generated by the dissolution heat of cement, the second exothermic peak by the hydration of C_4_A_3_$\bar{S}$, and the third exothermic peak by the secondary hydration of C_4_A_3_$\bar{S}$ [34]. However, the time and peak value of exothermic peaks are different between the two cements, especially for the second and third exothermic peaks. The time of the second and third exothermic peaks of W$\bar{S}$-GF-HBSAC-DG cement are 0.93 and 2.84 h, respectively, which are earlier than that of GF-HBSAC cement, which are 1.95 and 7.97 h, respectively. The peak values of the second and third exothermic peaks of W$\bar{S}$-GF-HBSAC-DG cement are 0.015 and 0.006 W/g, respectively, which are larger than that of GF-HBSAC cement, which are 0.012 and 0.005 W/g, respectively. Considering that the two cements have similar particle size distribution, as shown in Figure 12, the effect of cement fineness on the hydration rate can be negligible, so the exothermic process mainly depends on the cement composition. According to the results of QXRD analysis of clinkers and the method of cement preparation in Section 3.1.1, the difference between the two cements lies in the form of CaSO_4_ composition and the content of β-C_2_S, while the hydration of β-C_2_S is very slow. Therefore, the difference in Figure 11a is supposed to be caused by the difference between residual CaSO_4_ and DG [32,33]. The above results show that the residual CaSO_4_ in clinker can prolong the duration of induction period and reduce the hydration exothermic rate in the early stage, especially within 8 h. As shown in Figure 11b, the cumulative heat released from GF-HBSAC cement is gradually higher than that of W$\bar{S}$-GF-HBSAC-DG cement after 9.33 h. At 72 h, the cumulative heat released from GF-HBSAC cement reaches 197 J/g, while that of W$\bar{S}$-GF-HBSAC-DG cement is only 161 J/g, which is due to the combination of residual CaSO_4_ in GF-HBSAC clinker and water to release heat.

#### 3.2.3. Effect of CaSO_4_ Type on Mechanical Strength

**Figure 13:**
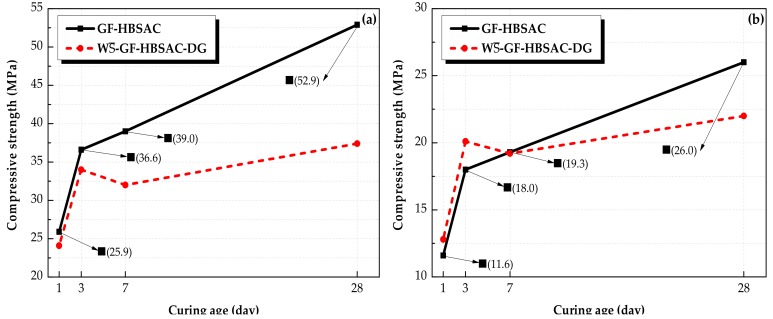
Compressive strength of the Series GF-HBSAC-X cement pastes under different water:cement ratios: (**a**) Water:cement ratio of 0.35; (**b**) water:cement ratio of 0.50.

**Figure 14:**
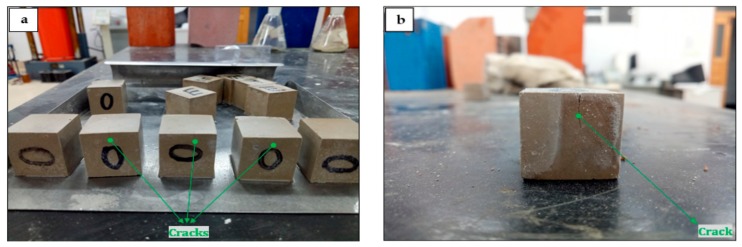
Development of cracks in W$\bar{S}$-GF-HBSAC-DG cement pastes with curing ages: (**a**) Seven days; (**b**) 28 days.

It can be seen from Figure 13 that the compressive strength of GF-HBSAC and W$\bar{S}$-GF-HBSAC-DG cement pastes under different water: cement ratios is comparatively consistent. There is little difference in the compressive strength between the two cement pastes during 1 to 3 days. Since 7 days, the compressive strength of GF-HBSAC cement pastes continuously increased, while that of W$\bar{S}$-GF-HBSAC-DG cement pastes showed a decrease in strength, which may be caused by the expansion and cracking of the cement pastes, as shown in Figure 14, and similar results have been found in [35], indicating that DG is likely to exceed the optimal content of gypsum. From this point of view, residual CaSO_4_ can completely replace DG to participate in hydration reaction. Compared with DG, residual CaSO_4_ can still ensure the stable development of cement strength when the design content of residual CaSO_4_ in clinker is 15%, reflecting the advantage of residual CaSO_4_ in HBSAC clinker based on solid wastes, at the same time, residual CaSO_4_ in clinker is also beneficial to the utilization of PCDS.

#### 3.2.4. Effect of CaSO_4_ Type on Hydration Products

**Figure 15:**
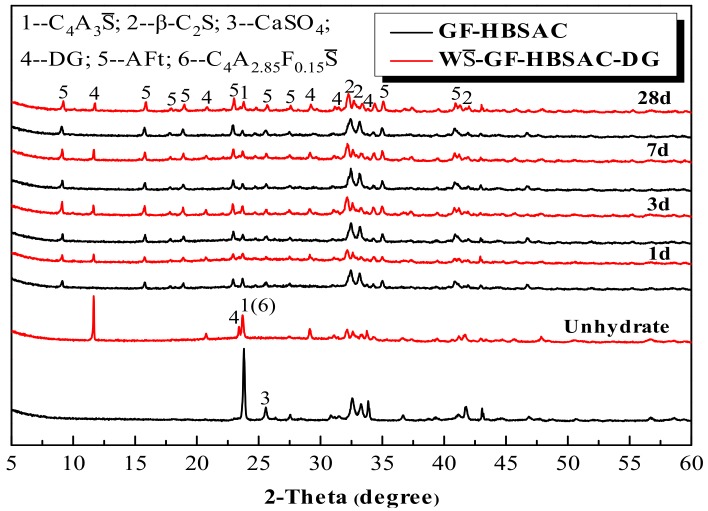
X-ray diffraction (XRD) patterns of the Series GF-HBSAC-X cement hydration products at different curing ages.

As can be seen from Figure 15, the development of hydration products of GF-HBSAC and W$\bar{S}$-GF-HBSAC-DG cement during 1 to 28 days is basically similar. In terms of the types of hydration products, the hydration products of both cements are mainly composed of AFt, indicating that residual CaSO_4_ can promote the hydration of C_4_A_3_$\bar{S}$ to form AFt as DG. From the formation process of hydration products, AFt formed in a large amount in both cements hydration at 1 day, while C_4_A_3_$\bar{S}$ and CaSO_4_ were consumed in large quantities. The diffraction peaks of C_4_A_3_$\bar{S}$ tended to be stable at 3 days and still existed at 28 days, and the diffraction peaks of β-C_2_S did not change significantly during the whole process, indicating that the hydration of C_4_A_3_$\bar{S}$ begins rapidly in the early stage, while the hydration of β-C_2_S is relatively slow, which is consistent with the viewpoint of [35,36].

**Figure 16:**
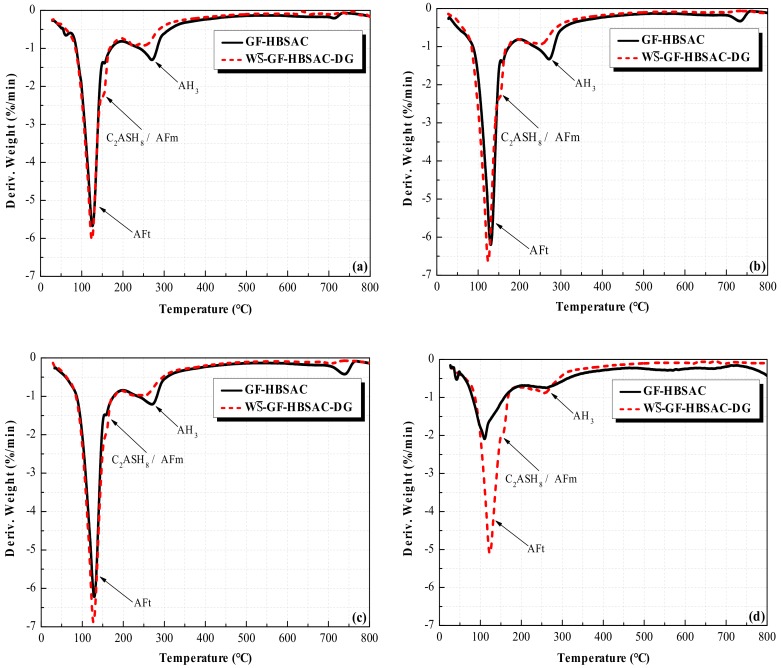
DTG curves of the Series GF-HBSAC-X cement hydration products at different curing ages: (**a**) One day; (**b**) 3 days; (**c**) 7 days; (**d**) 28 days.

As can be seen from Figure 16, the position of the dehydration peaks in DTG curves of GF-HBSAC and W$\bar{S}$-GF-HBSAC-DG cement is the same, indicating that the hydration products of the two cements are the same. Generally speaking, the dehydrated phase at 100–150 °C is AFt, the dehydrated phase at 130–160 °C is AFm or C_2_ASH_8_ (2CaO·Al_2_O_3_·SiO_2_·8H_2_O), and the dehydrated phase at 240–270 °C is AH_3_. The reason why AFm or C_2_ASH_8_ and AH_3_ are not reflected in XRD patterns is probably due to the low content and poor crystallization.

From the main hydration product, the AFt content of W$\bar{S}$-GF-HBSAC-DG cement hydration products is higher than that of GF-HBSAC cement during 1 to 28 days, which can be used to explain the development of cement pastes strength, as shown in Figure 13b and Figure 14. From other hydration products, the AH_3_ content of GF-HBSAC cement hydration products is higher than that of W$\bar{S}$-GF-HBSAC-DG cement, and the AFm or C_2_ASH_8_ content of GF-HBSAC cement hydration products is lower than that of W$\bar{S}$-GF-HBSAC-DG cement. According to the hydration reaction of Equations (1) and (2), the AH_3_ content is supposed to increase with the increase of AFt or AFm content, but now the decrease of AH_3_ content with the increase of AFt content means that AH_3_ may be further hydrated and consumed. The hydration reaction of Equation (6) has been mentioned in [35,36,37]. Now, the increase of AFm or C_2_ASH_8_ content with the decrease of AH_3_ content further confirms the possibility of this hydration reaction. Therefore, compared with the residual CaSO_4_ in clinker, the additional DG is more likely to promote the hydration of C_2_S.

C_2_S + AH_3_ + 5H → C_2_ASH_8_. (6)

**Figure 17:**
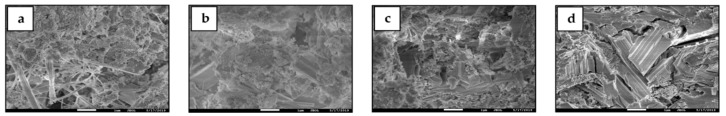
SEM micromorphology of GF-HBSAC cement hydration products at different curing ages: (**a**) One day; (**b**) 3 days; (**c**) 7 days; (**d**) 28 days.

**Figure 18:**
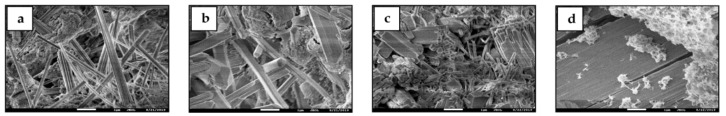
SEM micromorphology of W$\bar{S}$-GF-HBSAC-DG cement hydration products at different curing ages: (**a**) One day; (**b**) 3 days; (**c**) 7 days; (**d**) 28 days.

Figure 17 and Figure 18 show the SEM micromorphology of the two cements hydration products at different curing ages. According to the comparison, the two cements hydration products are basically the same, which are composed of needle-shaped AFt and cotton-shaped gel (including AH_3_, C-S-H, etc.). The gel is wrapped around AFt and plays the role of caulking and gluing, and the structure of AFt combined with gel provides the necessary strength for cement pastes. With the hydration reaction proceeding, AFt gradually develops from fine needle to thick rod. During 1 to 28 days, the AFt of W$\bar{S}$-GF-HBSAC-DG cement hydration products seems to be stronger than that of GF-HBSAC cement hydration products, and fine needle-shaped AFt appears in the W$\bar{S}$-GF-HBSAC-DG cement hydration products at 7 days, which is initially determined as the newly formed AFt at the expansion cracking site. Therefore, it can be seen that the development of hydration products, especially the formation of AFt, coincides with the development of strength.

### 3.3. Effect of CaSO_4_ Content on Properties of HBSAC Based on Solid Wastes

#### 3.3.1. Effect of CaSO_4_ Content on Water Requirement of Normal Consistency and Setting Time

**Figure 19:**
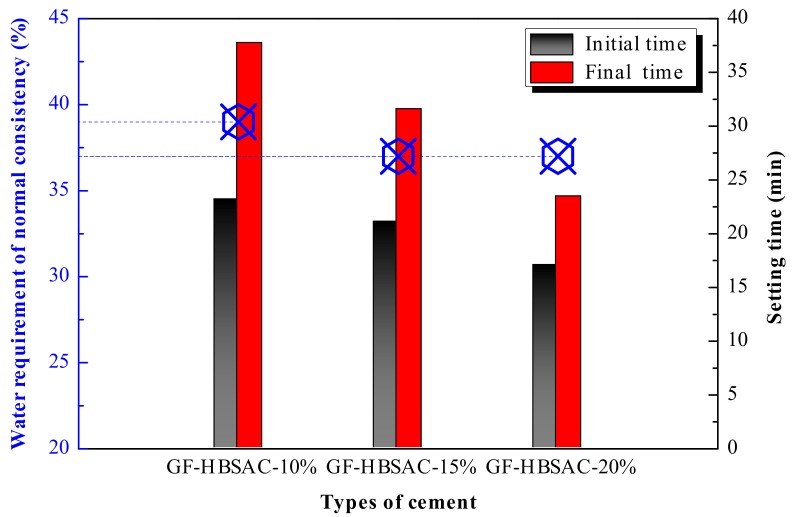
Water requirement of normal consistency and setting time of the Series GF-HBSAC-Y cement.

From Figure 19 it can be seen that, with the increase of residual CaSO_4_ content in clinker, the water requirement of normal consistency tends to decrease, and the setting time tends to shorten. It is well known that the effect of CaSO_4_ content on the water requirement of normal consistency and setting time is related to the hydration of C_4_A_3_$\bar{S}$ and the formation of AFt. With the increase of CaSO_4_ content, the hydration speed of C_4_A_3_$\bar{S}$ is accelerated [35,38], and the formation of AFt is also accelerated, which leads to the reduction of water demand and shortening of setting time. However, excessive CaSO_4_ content may cause the formation of AFt too fast and too much so that AFt wraps on the surface of clinker mineral particles and affects the hydration reaction to continue, which results in the reverse change of increasing water demand and prolonging setting time. In this study, the water requirement of normal consistency and setting time do not change in reverse regularity when the CaSO_4_ content increases, indicating that the increase of CaSO_4_ content within the design content range of 10%–20% is always conducive to the hydration of C_4_A_3_$\bar{S}$.

#### 3.3.2. Effect of CaSO_4_ Content on Hydration Heat

**Figure 20:**
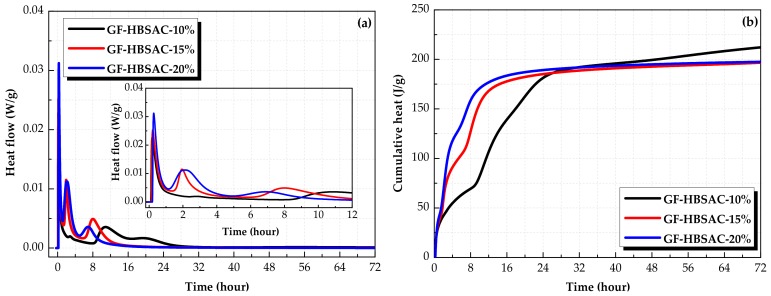
Hydration heat curves of the Series GF-HBSAC-Y cement. (**a**) Heat flow curve; (**b**) cumulative heat curve.

Figure 20 shows the hydration heat curves of the Series GF-HBSAC-Y cement. As can be seen from Figure 20a, the characteristics of heat flow curves of the three cements are similar, but the time and peak value of exothermic peaks are different. The first exothermic peaks of all three cements occur in about 10 min, and the peak value increases with the increase of CaSO_4_ content in clinker. For the second exothermic peak, the time appears earlier with the increase of CaSO_4_ content, and the duration and peak value increases with the increase of CaSO_4_ content. As for the third exothermic peak, the difference between the three cements is more obvious. With the increase of CaSO_4_ content, the time of exothermic peak appears earlier substantially, and the peak value increases first and then decreases, and the duration decreases gradually. It is concluded that the increase of CaSO_4_ content promotes the hydration of C_4_A_3_$\bar{S}$. Figure 18b shows the cumulative heat curve of GF-HBSAC cement with different CaSO_4_ contents. In the range of 0–26 h, the cumulative heat of three cements is as follows: GF-HBSAC-20% > GF-HBSAC-15% > GF-HBSAC-10%. In the range of 26–32 h, the cumulative heat of the three cements is as follows: GF-HBSAC-20% > GF-HBSAC-10% > GF-HBSAC-15%. After 32 h, the cumulative heat of three cements is as follows: GF-HBSAC-10% > GF-HBSAC-20% > GF-HBSAC-15%. It can be seen that the increase of residual CaSO_4_ content is beneficial to the hydration of C_4_A_3_$\bar{S}$ in the early stage of hydration, but it also brings larger hydration heat. With the hydration reaction proceeding, the hydration degree of GF-HBSAC-10% cement which reacts slowly in the early stage increases, and the cumulative heat increases to the highest at 3 days, while that of GF-HBSAC-15% and GF-HBSAC-20% cement are basically the same.

Based on the above analysis, GF-HBSAC-15% cement can not only maintain a good hydration rate, but also release less heat, which is the most prominent among the three cements with different CaSO_4_ contents.

#### 3.3.3. Effect of CaSO_4_ Content on Mechanical Strength

**Figure 21:**
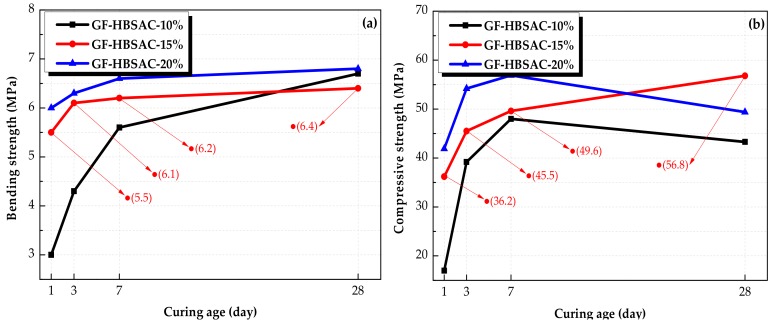
Mechanical strength of the Series GF-HBSAC-Y cement at different curing ages: (**a**) Bending strength; (**b**) compressive strength.

Figure 21a shows the development of bending strength of the Series GF-HBSAC-Y cement. From this figure, it can be seen that the bending strength increases with the increase of CaSO_4_ content during 1 to 7 days, but it no longer follows the same development rule during 7 to 28 days, and the relationship between the bending strength of three cements is GF-HBSAC-20% > GF-HBSAC-10% > GF-HBSAC-15%. The reason is that the strength growth is mainly due to the hydration of C_4_A_3_$\bar{S}$ to form AFt within 7 days, and the increase of CaSO_4_ content can accelerate the hydration rate of C_4_A_3_$\bar{S}$. During 7 to 28 days, for GF-HBSAC-20% and GF-HBSAC-15% cement, the hydration of C_4_A_3_$\bar{S}$ basically ended. In addition, the strength growth is dominated by the hydration of β-C_2_S in this stage. Therefore, the bending strength of GF-HBSAC-10% cement increases fastest.

Figure 21b shows the development of compressive strength of the Series GF-HBSAC-Y cement. From this figure, it can be seen that the development rule of compressive strength with CaSO_4_ content is the same as that of bending strength during 1 to 7 days, but there is a new development rule during 7 to 28 days. The compressive strength of both GF-HBSAC-10% and GF-HBSAC-20% cement shows a tendency of retrogression, and the relationship between the compressive strength of three cements is GF-HBSAC-15% > GF-HBSAC-20% > GF-HBSAC-10%. The reason is that the strength growth is mainly due to the hydration of C_4_A_3_$\bar{S}$ to form AFt within 7 days, and the increase of CaSO_4_ content can accelerate the hydration rate of C_4_A_3_$\bar{S}$. During 7 days to 28 days, the decrease in strength of GF-HBSAC-10% cement may be attributed to the formation of AFm [5,16,39], while the decrease in strength of GF-HBSAC-20% cement may be due to the micro-cracks, which are caused by the pressure of AFt crystals formed rapidly in the early stage continuous growth, and the pressure of gel filling cement paste pore in the later stage [35,40,41].

Based on the above analysis, the mechanical strength of GF-HBSAC-15% cement develops steadily, which is the most outstanding among the three cements.

#### 3.3.4. Effect of CaSO_4_ Content on Hydration Products

**Figure 22:**
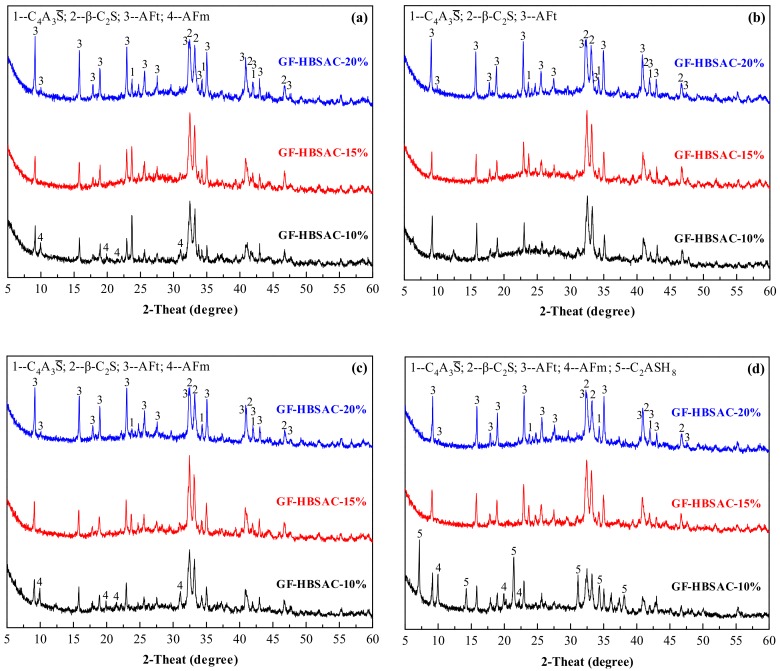
X-ray diffraction (XRD) patterns of the Series GF-HBSAC-Y cement hydration products at different curing ages: (**a**) One day; (**b**) 3 days; (**c**) 7 days; (**d**) 28 days.

It can be seen from Figure 22 that the main hydration product of GF-HBSAC-Y cement is AFt. At 1 day, with the increase of CaSO_4_ content, the diffraction peaks of C_4_A_3_$\bar{S}$ weaken and the diffraction peaks of AFt enhance, indicating that the increase of CaSO_4_ content promotes the hydration of C_4_A_3_$\bar{S}$ to form AFt. AFm appears in GF-HBSAC-10% cement, which should be the reaction of C_4_A_3_$\bar{S}$ with water to form low sulfur ettringite. At 3 days, for all three cements, the diffraction peaks of C_4_A_3_$\bar{S}$ continue to weaken and the diffraction peaks of AFt continue to enhance. During 3 to 7 days, the diffraction peaks of GF-HBSAC-10% cement hydration products vary the most, indicating that the hydration degree of GF-HBSAC-10% cement begins to increase. At 7 days, the diffraction peaks of GF-HBSAC-15% and GF-HBSAC-20% cement hydration products have not changed much, but for the GF-HBSAC-10% cement, the diffraction peaks of AFt begin to decrease, and the diffraction peaks of AFm reappear, which is considered to be transformed from AFt under the condition of low CaSO_4_ content. At 28 days, the diffraction peaks of AFm continue to enhance in GF-HBSAC-10% cement, which may lead to the weakening of cement pastes and the retrogression of strength. In addition, the diffraction peaks of β-C_2_S decrease considerably, and C_2_ASH_8_ formed in GF-HBSAC-10% cement, which should be the hydration product of reaction between β-C_2_S and AH_3_ according to Equation (6), but not observed in hydration products of the other two cements. Considering that the β-C_2_S content in GF-HBSAC-10% cement is the highest, this phenomenon may be related to the β-C_2_S content in cement.

**Figure 23:**
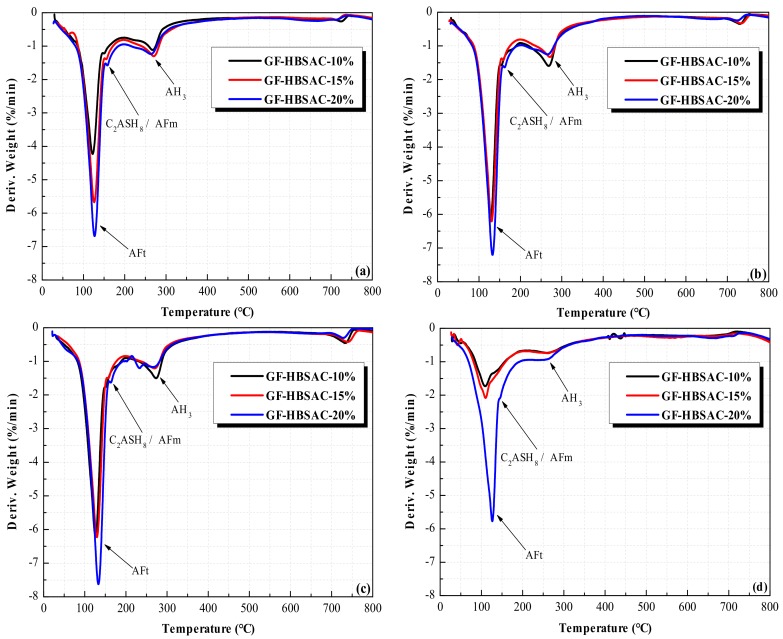
DTG curves of the Series GF-HBSAC-Y cement hydration products at different curing ages: (**a**) One day; (**b**) 3 days; (**c**) 7 days; (**d**) 28 days.

As can be seen from Figure 23, the position of the dehydration peaks in DTG curves of three cements is the same, indicating that the hydration products of three cements are the same. The hydration products are mainly composed of AFt, AFm, or C_2_ASH_8_ and AH_3_. For AFt, the content increases with the increase of CaSO_4_ content during 1 to 28 days, which is coincided with the change rule reflected by XRD patterns and further indicates that the increase of CaSO_4_ content promotes the hydration of C_4_A_3_$\bar{S}$. For AH_3_, the change rule with CaSO_4_ content varies in different curing ages. At 1 day, the AH_3_ content increases first and then decreased with the increase of CaSO_4_ content. Considering that hydration reaction at this stage is dominated by hydration of C_4_A_3_$\bar{S}$, the increase of CaSO_4_ content should promote hydration of C_4_A_3_$\bar{S}$ to form more AH_3_, but the AH_3_ content of GF-HBSAC-20% cement decreases instead, which should be caused by the consumption of part of AH_3_. Combining Equation (6), it can be considered that β-C_2_S in cement of high CaSO_4_ content takes the lead in hydration. During 3 to 7 days, the hydration degree of GF-HBSAC-10% cement increases, while the hydration of the other two cements slows down, so the AH_3_ content in GF-HBSAC-10% cement reached the highest level. In this stage, the reason why the diffraction peaks of hydration products such as C_2_ASH_8_ do not appear in XRD patterns may be related to the low hydration degree of β-C_2_S and the low crystallization degree of C_2_ASH_8_. At 28 days, the AH_3_ content of the three cements decreases, and the AH_3_ content of GF-HBSAC-10% cement decreases to the lowest, which may be due to the greatest hydration degree of β-C_2_S and the best crystallization degree of C_2_ASH_8_ in GF-HBSAC-10% cement, corresponding to the presence of C_2_ASH_8_ detected in XRD analysis. For AFm or C_2_ASH_8_, since the dehydration peaks of the two hydration products overlap, it is difficult to make a clear change rule of each product with CaSO_4_ content through DTG curves, which needs further study.

**Figure 24:**
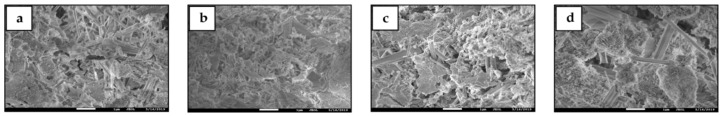
SEM micromorphology of GF-HBSAC-10% cement hydration products at different curing ages: (**a**) One day; (**b**) 3 days; (**c**) 7 days; (**d**) 28 days.

**Figure 25:**
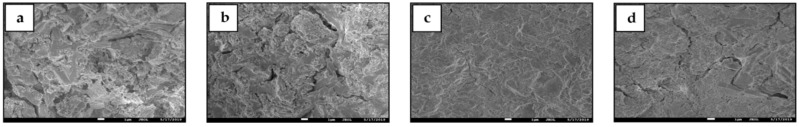
SEM micromorphology of GF-HBSAC-20% cement hydration products at different curing ages: (**a**) One day; (**b**) 3 days; (**c**) 7 days; (**d**) 28 days.

Figure 24 and Figure 25 show SEM micromorphology of GF-HBSAC-10% cement and GF-HBSAC-20% cement hydration products at different curing ages, respectively. Combined with Figure 17, for the three cements, the types of hydration products are basically similar, but the development of hydration products varies with different CaSO_4_ contents. For example, the AFt in GF-HBSAC-20% cement has developed into a thick rod and the structure of cement paste is relatively dense at 1 day, while the AFt in the other two cements is thin needles and the structure of cement paste is relatively sparse. Besides, lamellar AFm is found in the hydration products of GF-HBSAC-10% cement at 1 and 7 days, which is consistent with the results of XRD analysis. In the hydration products of GF-HBSAC-20% cement at 28 days, cracks around AFt can be clearly found, which should be caused by the expansion pressure because of the development of AFt. 

## 4. Conclusions

It is feasible to prepare HBSAC cement containing residual CaSO_4_ or no residual CaSO_4_ by using solid wastes including PCDS, FA, CS, and BX. The utilization rate of solid wastes in raw materials can reach more than 80%. The application provides a new channel for the utilization of various industrial solid wastes, especially for the utilization of PCDS.

Residual CaSO_4_ in clinker can take part in hydration instead of additional DG. Compared with additional DG, the residual CaSO_4_ in clinker has both advantages and disadvantages on the properties of HBSAC cement based on solid wastes. In the hydration process, the residual CaSO_4_ does not change the hydration products of HBSAC cement, and shows some advantages in strength. However, it increases the water requirement of normal consistency and hydration heat of cement, and prolongs the setting time. In addition, the promotion of residual CaSO_4_ on the hydration of β-C_2_S is not as strong as that of DG.

The residual CaSO_4_ content affects the calcination of HBSAC cement clinkers based on solid wastes. With the increase of CaSO_4_ content, the lower limit temperature of clinker formation increases gradually, but the optimal calcination temperature is not affected which is basically maintained at 1300 °C. The error between the actual and designed mineral content of clinkers prepared at 1300 °C is within a reasonable range. The micromorphology of clinkers also varies with the CaSO_4_ content. Meanwhile, the crystal size of clinker becomes finer and the boundary between crystals becomes more blurred.

The residual CaSO_4_ content also affects the hydration properties of HBSAC cement clinkers based on solid wastes. With the increase of CaSO_4_ content, the water requirement of normal consistency decreases, the setting time shortens, and the hydration heat releases in advance. In addition, the bending strength and compressive strength enhance with the increase of CaSO_4_ content during 1 to 7 days, but the bending strength of GF-HBSAC-10% cement still develops well during 7 to 28 days while that of the other two cements was basically unchanged, and the compressive strength of GF-HBSAC-15% cement shows a continuous development while that of the other two cements shows a retrogression. As for cement hydration products, the increase of CaSO_4_ content does not change the types of major hydration products, and AFt develops better with the increase of CaSO_4_ content. Meanwhile, AFm, as the secondary hydration product, is easy to produce in the process of cement hydration with lower CaSO_4_ content. Besides, the increase of CaSO_4_ content seems to promote the hydration of β-C_2_S, which will consume part of AH_3_ to form C_2_ASH_8_, but C_2_ASH_8_ has the better crystallization in cement with high β-C_2_S content and low CaSO_4_ content. Considering all aspects of analysis, the optimal design residue of CaSO_4_ is 15%.

## Figures and Tables

**Table 1:** Chemical composition of the raw materials, wt.%.

Raw Material	CaO	Al_2_O_3_	SiO_2_	Fe_2_O_3_	SO_3_	MgO	TiO_2_	LOI	∑
PCDS	52.93	0.96	4.56	1.13	30.12	2.03	0.00	7.48	99.21
FA	7.86	27.45	52.56	4.24	1.26	1.12	0.99	1.73	97.21
CS	66.02	1.47	4.61	0.68	1.97	0.25	0.00	24.62	99.62
BX	0.51	64.07	14.53	0.88	0.00	15.38	2.56	1.03	98.96
DG	32.56	/	/	/	46.51	/	/	20.92	99.99

**Table 2:** Design of the mineral composition of the clinkers and the proportion of the raw materials, wt.%.

**Series**	**Cement**	**Preliminary ClinkerMinerals**				**Raw Materials**			
		**C_4_AF**	**C_4_A_3_**$\bar{S}$	**C_2_S**	**CaSO_4_**	**PCDS**	**FA**	**CS**	**BX**
GF-HBSAC-X	W$\bar{S}$-GF-HBSAC-DG	5	42	53	0	14.7	16.9	49.2	19.2
	GF-HBSAC	5	35	45	15	37.5	14.3	32.0	16.2
GF-HBSAC-Y	GF-HBSAC-15%								
	GF-HBSAC-10%	5	35	50	10	29.0	16.0	38.6	16.4
	GF-HBSAC-20%	5	35	40	20	47.2	9.9	23.2	19.7
**Series**	**Cement**		**Final Clinker Minerals**						
			**C_4_AF**		**C_4_A_3_**$\bar{S}$	**C_2_S**	**CaSO_4_**		**CaO**
GF-HBSAC-X	W$\bar{S}$-GF-HBSAC-DG		5.27		42.15	52.47	0		0.11
	GF-HBSAC		5.09		34.24	45.14	15.45		0.08
GF-HBSAC-Y	GF-HBSAC-15%								
	GF-HBSAC-10%		5.23		36.12	48.52	10.08		0.05
	GF-HBSAC-20%		4.63		36.25	38.38	20.68		0.06

Note: The amount of PCDS in the table does not include any additional parts due to decomposition.

**Table 3:** Chemical composition and the three moduli of the Series GF-HBSAC-X cement clinkers.

Clinker	Chemical Composition (%)								The Three Moduli		
	CaO	Al_2_O_3_	SiO_2_	Fe_2_O_3_	SO_3_	MgO	TiO_2_	∑	*C_m_*	*P*	*N*
GF-HBSAC	46.75	18.24	14.13	2.47	12.93	2.30	0.60	97.42	1.02	1.41	1.29
W$\bar{S}$-GF-HBSAC-DG	48.06	21.91	16.77	2.52	5.82	2.52	0.71	97.84	1.00	3.76	1.31

**Table 4:** Mineral composition of the Series GF-HBSAC-X cement clinkers.

Clinker	Mineral Composition (%)			
	$C_{4}A_{3}\bar{S}$	β-C_2_S	CaSO_4_	Others
GF-HBSAC	36.27	50.96	12.62	0.15
W$\bar{S}$-GF-HBSAC-DG	43.18	56.53	0.00	0.29

**Table 5:** Chemical composition and the three moduli of the Series GF-HBSAC-Y cement clinkers.

Clinker	Chemical Composition (%)								The Three Moduli		
	CaO	Al_2_O_3_	SiO_2_	Fe_2_O_3_	SO_3_	MgO	TiO_2_	∑	*C_m_*	*P*	*N*
GF-HBSAC-10%	47.23	18.09	15.46	2.44	9.86	2.42	0.64	96.14	1.02	1.83	1.17
GF-HBSAC-15%	46.75	18.24	14.13	2.47	12.93	2.30	0.60	97.42	1.02	1.41	1.29
GF-HBSAC-20%	45.83	18.25	12.63	2.15	15.88	3.00	0.77	98.51	1.01	1.15	1.44

**Table 6:** Mineral composition of the Series GF-HBSAC-Y cement clinkers.

Clinker	Mineral Composition (%)			
	$C_{4}A_{3}\bar{S}$	β-C_2_S	CaSO_4_	Others
GF-HBSAC-10%	36.13	55.28	8.34	0.25
GF-HBSAC-15%	36.27	50.96	12.62	0.15
GF-HBSAC-20%	35.91	46.36	17.65	0.08

## Abbreviations

The following abbreviations are used in this manuscript:

HBSAC	high belite sulfoaluminate cement
SAC	sulfoaluminate cement
C$\bar{S}$	CaSO_4_
C$\bar{S}$H_2_	CaSO_4_·2H_2_O
C_4_A_3_$\bar{S}$	3CaO·3Al_2_O_3_·CaSO_4_
β-C_2_S	2CaO·SiO_2_
C_5_S_2_$\bar{S}$	4CaO·2SiO_2_·CaSO_4_
C_4_AF	4CaO·Al_2_O_3_·Fe_2_O_3_
f-CaO	free CaO
C_4_A_2.85_F_0.15_$\bar{S}$	3CaO·2.85Al_2_O_3_·0.15Fe_2_O_3_·CaSO_4_
C_2_ASH_8_	2CaO·Al_2_O_3_·SiO_2_·8H_2_O
AH_3_	Al_2_O_3_·3H_2_O
AFt	3CaO·Al_2_O_3_·3CaSO_4_·32H_2_O
AFm	3CaO·Al_2_O_3_·CaSO_4_·12H_2_O
PCDS	petroleum coke desulfurization slag
FA	fly ash
CS	carbide slag
BX	bauxite
DG	dihydrate gypsum
